# Comparative analysis of BERT-based and generative large language models for detecting suicidal ideation: a performance evaluation study

**DOI:** 10.1590/0102-311XEN028824

**Published:** 2024-11-25

**Authors:** Adonias Caetano de Oliveira, Renato Freitas Bessa, Ariel Soares Teles

**Affiliations:** 1 Instituto Federal de Educação, Ciência e Tecnologia do Ceará, Fortaleza, Brasil.; 2 Universidade Federal do Delta do Parnaíba, Parnaíba, Brasil.; 3 Instituto Federal do Maranhão, São Luís, Brasil.

**Keywords:** Suicide, Suicidal Ideation, Artificial Intelligence, Natural Language Processing, Suicídio, Ideação Suicida, Inteligência Artificial, Processamento de Linguagem Natural, Suicidio, Ideación Suicida, Inteligencia Artificial, Procesamiento de Lenguaje Natural

## Abstract

Artificial intelligence can detect suicidal ideation manifestations in texts. Studies demonstrate that BERT-based models achieve better performance in text classification problems. Large language models (LLMs) answer free-text queries without being specifically trained. This work aims to compare the performance of three variations of BERT models and LLMs (Google Bard, Microsoft Bing/GPT-4, and OpenAI ChatGPT-3.5) for identifying suicidal ideation from nonclinical texts written in Brazilian Portuguese. A dataset labeled by psychologists consisted of 2,691 sentences without suicidal ideation and 1,097 with suicidal ideation, of which 100 sentences were selected for testing. We applied data preprocessing techniques, hyperparameter optimization, and hold-out cross-validation for training and testing BERT models. When evaluating LLMs, we used zero-shot prompting engineering. Each test sentence was labeled if it contained suicidal ideation, according to the chatbot’s response. Bing/GPT-4 achieved the best performance, with 98% across all metrics. Fine-tuned BERT models outperformed the other LLMs: BERTimbau-Large performed the best with a 96% accuracy, followed by BERTimbau-Base with 94%, and BERT-Multilingual with 87%. Bard performed the worst with 62% accuracy, whereas ChatGPT-3.5 achieved 81%. The high recall capacity of the models suggests a low misclassification rate of at-risk patients, which is crucial to prevent missed interventions by professionals. However, despite their potential in supporting suicidal ideation detection, these models have not been validated in a patient monitoring clinical setting. Therefore, caution is advised when using the evaluated models as tools to assist healthcare professionals in detecting suicidal ideation.

## Resumo

A inteligência artificial pode detectar manifestações de ideação suicida em textos. Estudos demonstram que os modelos baseados em BERT alcançam melhor desempenho em testes de classificação de texto. Os grandes modelos de linguagem (LLMs - *large language models*) respondem a consultas de texto livre sem serem especificamente treinados. Este trabalho tem como objetivo comparar o desempenho de três variações de modelos BERT e LLMs (Google Bard, Microsoft Bing/GPT-4 e OpenAI ChatGPT-3.5) para identificar ideação suicida a partir de textos não clínicos escritos em Português brasileiro. Foi usado um conjunto de dados rotulado por psicólogos composto por 2.691 sentenças sem ideação suicida e 1.097 com ideação suicida, das quais 100 sentenças foram selecionadas para o processo de teste. Técnicas de pré-processamento de dados, otimização de hiperparâmetros e validação cruzada *holdout* foram aplicadas para treinar e testar os modelos BERT. Ao avaliar LLMs, usamos comandos de disparo zero. Cada frase de teste foi rotulada com base na presença de ideação suicida, de acordo com a resposta do *chatbot*. O Bing/GPT-4 alcançou o melhor desempenho, demonstrando 98% em todas as métricas. Os modelos BERT ajustados superaram os outros LLMs: o BERTimbau-Large teve o melhor desempenho, demonstrando 96% de acurácia, seguido pelo BERTimbau-Base com 94% e pelo BERT-Multilingual com 87%. O Bard teve o pior desempenho, apontando 62% de acurácia, enquanto o ChatGPT-3.5 alcançou 81%. O alto *recall* dos modelos indica uma baixa taxa de falsos negativos de pacientes em risco, o que é crucial para evitar intervenções profissionais desnecessárias. No entanto, apesar de seu potencial no suporte à detecção de ideação suicida, esses modelos não foram validados em um ambiente clínico de monitoramento de pacientes. Portanto, recomenda-se cautela ao empregar esses modelos como ferramentas para auxiliar profissionais de saúde na detecção de ideação suicida.

## Resumen

La inteligencia artificial puede detectar manifestaciones de ideación suicida en textos. Los estudios demuestran que los modelos basados en BERT logran un mejor rendimiento en las pruebas de clasificación de textos. Los grandes modelos de lenguaje (LLMs, *large language models*) responden a consultas de texto libre sin estar específicamente capacitados. Este trabajo tiene como objetivo comparar el rendimiento de tres variaciones de modelos BERT y LLMs (Google Bard, Microsoft Bing/GPT-4 y OpenAI ChatGPT-3.5) para identificar ideación suicida con base en textos no clínicos escritos en Portugués brasileño. Se utilizó un conjunto de datos etiquetados por psicólogos que constaba de 2.691 sentencias sin ideación suicida y 1.097 con ideación suicida, de las cuales se seleccionaron 100 sentencias para el proceso de prueba. Técnicas de preprocesamiento de datos, optimización de hiperparámetros y validación cruzada *holdout* se aplicaron para entrenar y probar modelos BERT. Al evaluar los LLM, utilizamos comandos de disparo cero. Cada frase de prueba fue etiquetada con base en la presencia de ideación suicida, según la respuesta del *chatbot*. Bing/GPT-4 logró el mejor rendimiento, demostrando un 98% en todas las métricas. Los modelos BERT ajustados superaron a los otros LLM: BERTimbau-Large obtuvo el mejor rendimiento, demostrando un 96% de *accuracy*, seguido de BERTimbau-Base con un 94% y de BERT-Multilingual con un 87%. Bard tuvo el peor rendimiento, logrando un 62% de *accuracy*, mientras que ChatGPT-3.5 logró un 81%. El alto *recall* de los modelos indica una baja tasa de falsos negativos de pacientes en riesgo, lo cual es crucial para evitar intervenciones profesionales innecesarias. Sin embargo, a pesar de su potencial para respaldar la detección de ideación suicida, estos modelos no se han validado en un entorno clínico de seguimiento de pacientes. Por lo tanto, se recomienda precaución al emplear estos modelos como herramientas para ayudar a los profesionales de la salud a detectar ideación suicida.

## Introduction

According to the World Health Organization (WHO) ^1^, more than 700,000 people commit suicide every year, that is, one death every 40 seconds. Among adolescents and young people, suicide was the fourth leading cause of death for individuals aged 15 to 29 worldwide in 2019. From 2000 to 2017, Brazil experienced a 75% increase in suicide deaths for men and 85% for women ^2^. In 2019, Brazil was among the top 10 countries where the most suicides occurred in the world, the second among countries of the Americas ^1^. Suicide is considered a very challenging global public health issue. It is difficult to predict since it is influenced by multiple factors, such as biological, psychological, and genetic conditions, economic recessions in the country, media coverage of suicide, and environmental, financial, social, and even cultural situations ^3^.

Among the advances in artificial intelligence (AI) and natural language processing (NLP), the excellent performance of language models (LMs) stands out, which has been achieved in solving several complex tasks via text processing. LMs are AI models built on an architecture of varying complexity, from simple models to more robust neural network models with numerous parameters ^4^^,^^5^^,^^6^. Large language models (LLMs) are transformer language models ranging from millions to hundreds of billions (even trillions) of parameters, trained on massive text data and with exceptional learning capacity to handle sequential data efficiently, enabling parallelization and capturing long-term dependencies reached in the text ^4^^,^^7^^,^^8^. Based on training from a prompt or context, generative LLMs can generate coherent and meaningful responses, making them suitable for interactive and conversational applications, such as sentiment/emotion analysis in medical applications ^7^^,^^8^^,^^9^^,^^10^.

In the evolution of LMs towards LLMs, another alternative LMs application has been opened in the healthcare domain to respond to free-text queries with specific professional knowledge ^10^^,^^11^. Some examples of applications in healthcare are supporting the preparation of clinical documentation, generation of discharge summaries, clinical, operational, and procedural notes, and use of the chatbot to answer patient questions with their specific data and concerns ^12^. LLMs have demonstrated an excellent scientific knowledge base in biology and medical examinations, beneficial for research and healthcare practice ^8^^,^^13^^,^^14^.

LLMs have been applied to classify texts, i.e., to assign labels to texts of different lengths, such as sentences, paragraphs, and documents ^13^. Promising results demonstrate that BERT-based models ^15^ perform well on text classification problems, such as identifying prescription drugs mentioned in tweets ^14^, classifying news, posts, and tweets about COVID-19 as true information or fake news ^16^, detection of depression ^17^^,^^18^, identification of self-harm, and suicidal ideation ^19^. Moreover, LLMs present an opportunity to improve just-in-time adaptive interventions via mobile devices (e.g., smartphones, tablet computers ^17^), which can remotely monitor patient texts. These interventions can provide support at an adequate time, in the context that a patient needs the most, and with a significant likelihood of being receptive ^20^. This approach can support mental health professionals (e.g., psychiatrists, psychologists) in their clinical or therapeutic decisions by remotely monitoring the level of suicide risk of their patients, including early detection, and applying more appropriate interventions ^21^^,^^22^^,^^23^.

With an appropriate training process, BERT-based models can identify suicidal ideation in texts ^17^^,^^21^^,^^24^. In the case of generative LLMs, as they are not specifically trained on the task, adequate prompt engineering is necessary to develop effective queries to check if texts contain suicidal ideation ^25^^,^^26^^,^^27^. LLMs, such as BERT-based and generative models, can be integrated into NLP-based suicide prevention support systems for remote patient monitoring ^21^, identification of suicidal ideation manifestations on digital platforms (e.g., mobile applications and social networks) ^17^^,^^28^^,^^29^, and automated diagnosis ^30^. Therefore, this complementary suicide prevention methodology aids mental health professionals identify crucial situations to perform early interventions in patients ^21^^,^^22^^,^^23^^,^^31^.

This study aims to compare the performance of three variations of BERT-based and generative LLMs (Microsoft Bing, OpenAI ChatGPT-3.5, and Google Bard) in zero-shot prompting for identifying suicidal ideation from non-clinical texts written in Brazilian Portuguese. We analyzed the performance of LLMs in detecting suicide risk situations via the suicidal ideation manifestation in texts compared to the BERT Multilingual and BERTimbau models (base and large).

## Materials and methods

**Figure 1:**
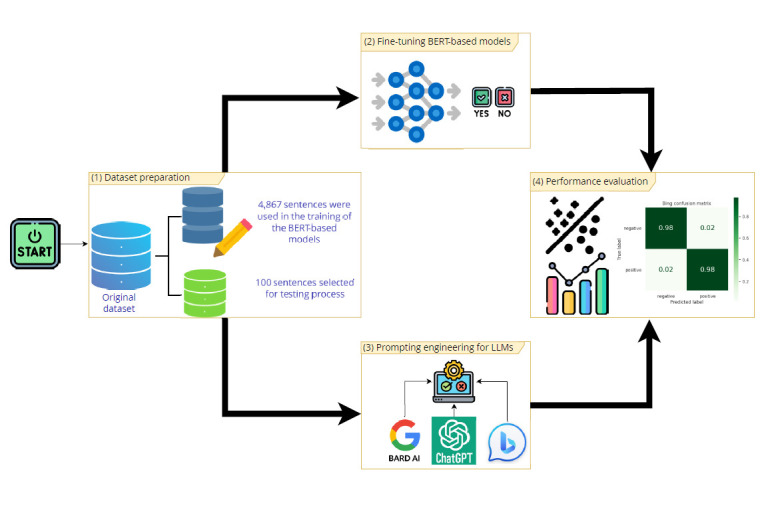
Methodological procedures performed in the study.

AI: artificial intelligence; LLMs: large language models.

The methodology of this study was organized into four stages, as shown in Figure 1 and detailed in the following sections.

### Dataset collection, annotation, and preparation

The data collection consisted of retrieving 5,699 tweets in Brazilian Portuguese from Twitter users. Tweets were downloaded using the Twitter API (https://developer.x.com/en/docs/x-api) in a customized manner depending on search sentences linked to suicide ^32^. Only the posts content remained after removing irrelevant information and user-specific data ^21^.

In total, three psychologists from different psychological approaches were invited to the data annotation process. They individually categorized each tweet as either positive (coded as 1) or negative (coded as 0) for suicidal ideation. After eliminating duplicates (n = 398) and samples that caused disagreements among psychologists (n = 1,513), the final dataset included 1,097 sentences labeled as positive and 2,691 labeled as negative ^21^. The dataset ^33^ is available in CSV format in two columns: text and target, corresponding to sentences and classes (0 or 1), respectively.

**Figure 2:**
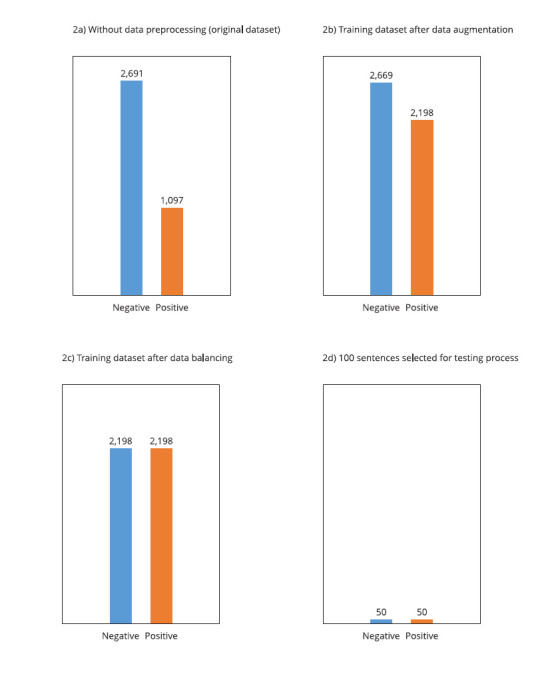
Number of instances labeled as negative and positive.

A total of 100 sentences (50 of each class) were selected from the original dataset to be used for testing the LLMs (Figure 2), which required no data preprocessing. However, to train and test BERT models, data preparation techniques were applied to obtain better performance and avoid bias. All the following techniques were applied to the training dataset, while only techniques 1, 2, and 3 were applied to the testing dataset.

(1) Lowercase conversion: conversion of all terms to lowercase to keep the flow consistent during NLP, since conversion aids to reduce the variability in text;

(2) Text cleaning: exclusion of decontextualized terms, such as social media aliases, email addresses, numbers, special symbols, and URLs;

(3) Removal of stop words: removal of some very frequent words that add minimal semantics;

(4) Data augmentation: performed by generating 1,151 synthetic sentences positive for suicidal ideation, as the dataset was unbalanced. The negative class had more sentences than the positive class (Figure 2). For this purpose, the *nlpaug* library was used ^34^;

(5) Data balancing: after data augmentation, the dataset was split, with 4,867 sentences used for training (Figure 2). The majority class (negative) was undersampled by randomly choosing sentences without replacement using the *RandomUnderSampler* of imbalanced-learn library ^35^ (Figure 2). This technique was necessary since, even after data augmentation, the dataset still showed an unbalanced training dataset with 2,198 positives and 2,669 negatives.

### Fine-tuning BERT-based models

In total, three BERT-based models were pretrained in Brazilian Portuguese, namely: BERTimbau-Base, BERTimbau-Large ^36^, and BERT-Multilingual ^15^. First, tokenization was conducted for encoding raw texts into tokens. To obtain the best-performing BERT models, the AdamW optimizer was used to adjust parameters in the model, with a batch size of 16, configured with a learning rate equal to 2e-6 in seven training epochs. Experiments included 3 to 8 epochs. Hold-out validation was performed by dividing the preprocessed dataset into 4,396 sentences for training and 100 for testing.

### Prompt engineering for generative LLMs

Prompt engineering involves creating prompts optimized to employ LLMs across multiple applications and research topics efficiently ^1^^,^^7^^,^^37^^,^^38^. Thus, a systematic input design is needed to obtain optimized prompts that guide the LLMs’ responses without losing coherence in the generated output and ensuring its accuracy and relevance ^7^^,^^37^^,^^39^. To make LLMs more accessible and applicable in different domains, the prompt engineering process is crucial to harness the full potential of the models ^40^. Thus, researchers can improve the capacity of LLMs in a wide range of common and complex tasks ^41^, such as answering questions to assess whether sentences contain suicidal ideation.

This study evaluated three generative LLMs: OpenAI ChatGPT-3.5, Google Bard, and Microsoft Bing Chat (Bing/GPT-4). They are based on the transformer-type model architecture that incorporates a self-attention mechanism, enabling the model to focus on various parts of the input sequence with varying levels of attention ^40^^,^^42^. Bing runs on GPT-4 ^40^ and, in this study, was defined to work on the “more precise” mode.

The zero-shot prompting approach was adopted, i.e., no examples were provided to the model in question prompts ^6^^,^^40^^,^^42^. Zero-shot prompting was selected due to the simplicity of this approach, with quality results when faced with domain-specific questions ^39^^,^^41^^,^^42^. Although the zero-shot prompting technique was adopted, the conversation was contextualized using the following question in Brazilian Portuguese: “Can you identify whether there is suicidal ideation in one sentence?”. Whenever the conversation session expired, this contextualization process was repeated.

For each sentence, the following structure was employed in Brazilian Portuguese: “<sentence>. Is there suicidal ideation in the sentence?”. Each sentence in the testing dataset was classified as positive or negative according to the chatbot’s explicit response. An unknown response was considered when the chatbot said it could not inform whether a sentence contained suicidal ideation. This occurred because, in some cases, chatbots indicated that additional context was required for the sentence, as it could be interpreted as positive or negative for suicidal ideation (i.e., ambiguity). These unknown responses were considered classification errors.

### Performance evaluation

Supplementary Material - Table S1

The testing sentences obtained from the original dataset were organized in a spreadsheet (Supplementary Material - Table S1; https://cadernos.ensp.fiocruz.br/static//arquivo/suppl-e00028824_1001.pdf) with the following data: a column referring to the sentence, a column for the actual class of the sentence, and columns to record the predicted class of each model. A confusion matrix (Box 1) was generated to estimate performance metrics from the following values.

**Box 1:** Confusion matrix.

		PREDICTED LABEL		
	Negative	Positive	Total
ACTUAL LABEL	Negative	True negative (TN)	False positive (FP)	TN + FP
	Positive	False negative (FN)	True positive (TP)	FN + TP
	Total	TN + FN	FP + TP	

True positive (TP): a sentence that the model correctly classifies as positive for suicidal ideation;

True negative (TN): a sentence that the model correctly classifies as negative for suicidal ideation;

False positive (FP): a sentence that the model incorrectly classifies as positive for suicidal ideation;

False negative (FN): a sentence that the model incorrectly classifies as negative for suicidal ideation.

The performance of the models was analyzed according to the following metrics ^43^: Accuracy (Equation 1); Precision (Equation 2); Recall (Equation 3); and F1-score (Equation 4). In addition, the area under the receiver operating characteristic curve (ROC-AUC) was computed.

$Accuracy=\frac{TP+TN}{TP+TN+FP+FN}$ (Equation 1)

$Precision=\frac{TP}{TP+FP}$ (Equation 2)

$Recall=\frac{TP}{TP+FN}$ (Equation 3)

$F1-score=\frac{2\times \left(Precision\times Recall\right)}{Precision+Recall}$ (Equation 4) 

### Study quality assessment

A questionnaire ^44^ was applied to assess the quality of this study. The questionnaire is a checklist composed of 30 items to qualitatively evaluate the contribution and reproducibility of results. There are three options for each item: “not applicable - NA”, “not addressed - No,” and “addressed - Yes”. The first author conducted the assessment, which was verified by the two others.

## Results

### Performance of the models

**Figure 3:**
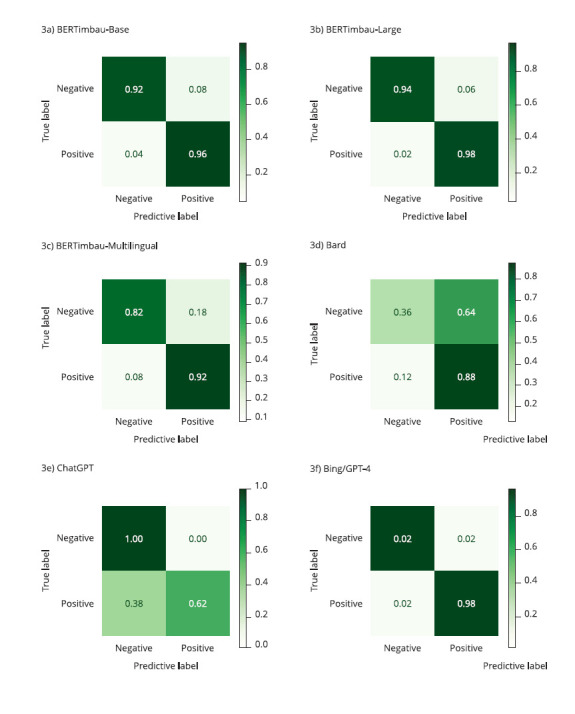
Performance of the models via confusion matrices: a visual representation of classification results, revealing the strengths and weaknesses of large language models (LLMs) in identifying suicidal ideation. Bing/GPT-4 achieved the best performance by classifying 98 sentences correctly.

Note: BERTimbau-Large correctly classified 96 sentences, followed by BERTimbau-Base (n = 94), BERT-Multilingual (n = 87), ChatGPT (n = 81), and Bard (n = 62). Only Bard (n = 15) and Bing/GPT-4 (n = 2) provided unknown responses.

Figure 3 displays the confusion matrices with classification results. Table 1 shows the performance results for the six models regarding accuracy, precision, recall, and F1-score. Bing/GPT-4 achieved the best accuracy and excellent results in other metrics. The fine-tuning BERTimbau models outperformed the other LLMs with accuracy ≥ 94%, followed by BERT-multilingual with 87%. ChatGPT achieved a 81% accuracy, whereas Bard performed worse by incorrectly classifying 23 sentences (62% accuracy).

**Table 1:** Performance results of the models.

Models	Classification	Accuracy (%)	Precision (%)	Recall (%)	F1-score (%)
BERTimbau-Base	Negative	94	92	96	94
	Positive		96	92	94
BERTimbau-Large	Negative	96	94	98	96
	Positive		98	94	96
BERT-Multilingual	Negative	87	82	91	86
	Positive		92	84	88
ChatGPT-3.5	Negative	81	100	72	84
	Positive		62	100	77
Bard	Negative	62	36	75	49
	Positive		88	58	70
Bing/GPT-4	Negative	98	98	98	98
	Positive		98	98	98

Regarding precision, we found that ChatGPT correctly classified all 50 sentences in the negative class; in other words, it indicates that this chatbot is quite efficient in identifying sentences that do not present suicidal ideation. Bing/GPT-4 performed similar to ChatGPT, with 49 correctly classified negative class sentences. BERTimbau models performed better in the positive class sentences, with 48 sentences correctly classified by BERTimbau-Base and 49 sentences by BERTimbau-Large (same performance as Bing). Moreover, we found the best recall result in ChatGPT for the positive class, followed by Bing/GPT-4 with just one incorrectly classified sentence. BERTimbau models had excellent recall for the negative class, with performance above 48 correctly classified sentences.

**Figure 4:**
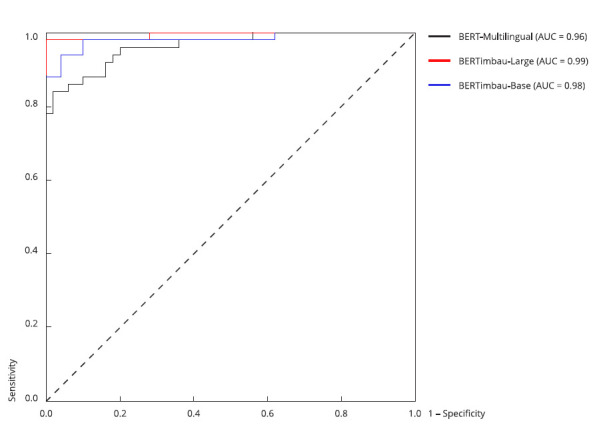
Performance comparison between BERT-based models using the receiver operating characteristic (ROC) curve.

Note: analysis of the BERTimbau-Large, BERTimbau-Base, and BERT-Multilingual variations highlights the differences in sensitivity and specificity in the classification. The ROC curve compares the models with the area under the receiver operating characteristic curve (ROC-AUC) determined by the confidence values versus the actual outputs. BERTimbau-Large has an AUC of 0.99, followed by BERTimbau-Base (AUC = 0.98) and BERT-multilingual (AUC = 0.96).

**Figure 5:**
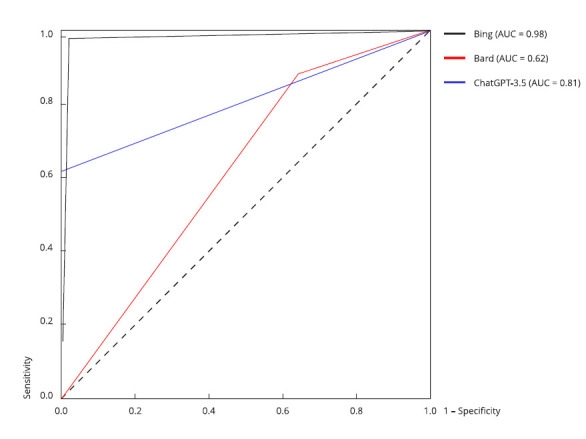
Performance comparison between generative models using the receiver operating characteristic (ROC) curve.

Note: analysis of the ChatGPT-3.5, Bing/GPT-4, and Bard models highlights the differences in sensitivity and specificity in the classification. In the case of generative large language models (LLMs), which do not provide confidence values, area under the receiver operating characteristic curve (ROC-AUC) is determined by the predicted outputs versus the actual outputs. Bing/GPT-4 shows an AUC of 0.96, followed by ChatGPT-3.5 (AUC = 0.81) and Bard (AUC = 0.62).

Figures 4 and 5 display the ROC-AUC plots of the BERT-based and generative LLMs, respectively. Figure 4 indicates that BERTimbau-Large (AUC = 0.99) shows the best overall capacity to distinguish sentences between classes, compared to BERTimbau-Base (AUC = 0.98) and BERT-multilingual (AUC = 0.96). Figure 5 indicates that Bing/GPT-4 (AUC = 0.96) shows high accuracy, with an excellent combination of sensitivity and specificity.

### Study quality

Supplementary Material -Table S2

**Figure 6:**
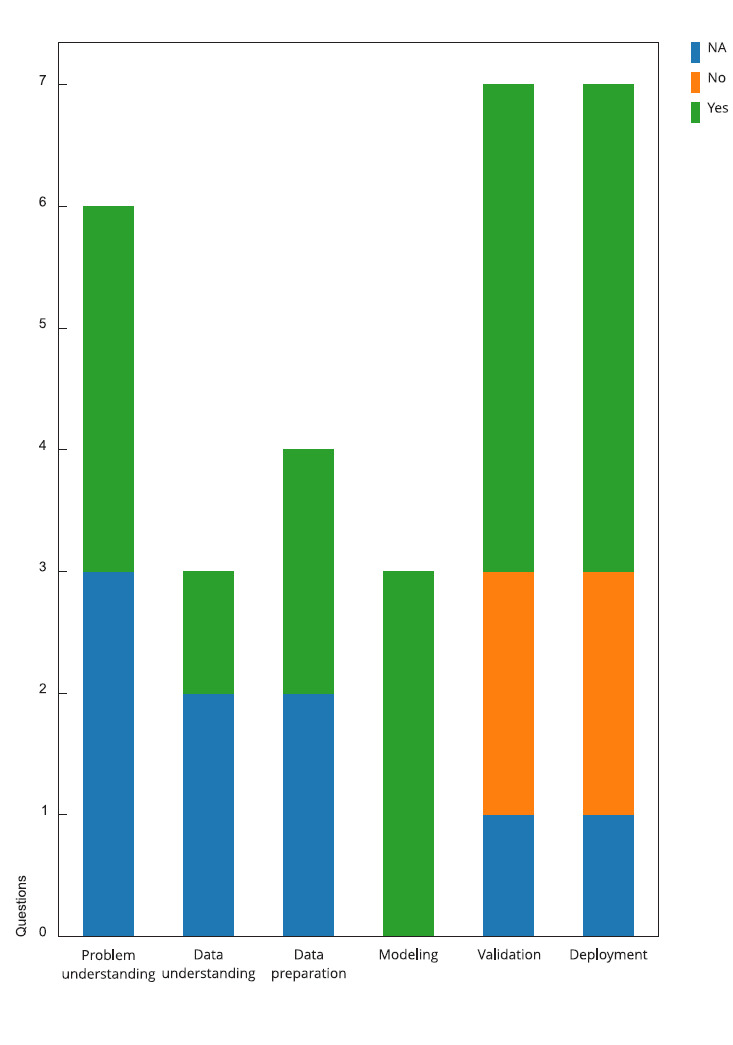
Results of the study quality assessment.

Figure 6 summarizes the responses (Supplementary Material - Table S2; https://cadernos.ensp.fiocruz.br/static//arquivo/suppl-e00028824_1001.pdf) to the quality assessment questionnaire ^44^ applied for this study. Each bar represents a study phase considered by the questionnaire.

NA: not applicable.

## Discussion

### Key results

ChatGPT achieved excellent performance for detecting people at risk of suicide (100% recall in suicidal ideation-positive sentences), followed by the Bing/GPT-4 with 98% and BERTimbau-Large with 94% recall. These results suggest that the sentences identified as positive for suicidal ideation by the ChatGPT, Bing/GPT-4, and BERTimbau-Large models were actually positive for suicidal ideation. With this lower rate of false positives, there is less chance of harmful situations occurring in which patients at risk of suicide are left without professional intervention. To effectively prevent suicide attempts, it is crucial to identify all individuals at risk, including those who may not initially appear so, enabling comprehensive analysis by professionals at a later stage.

The other results show that Bing/GPT-4 was the model that performed best in the task of identifying suicidal ideation in non-clinical texts in Brazilian Portuguese. The chatbot could not identify suicidal ideation in only two sentences. The fact that Bing is based on GPT-4 was a differentiator to other LLMs, as it presents improved multilingual capacities compared to ChatGPT-3.5 and Bard ^27^. Bing/GPT-4 was the best at balancing the trade-off between precision and recall with a 98% F1-score, although ChatGPT-3.5 was more precise in classifying negative class sentences and more sensitive in classifying positive class sentences. BERT-based models outperformed ChatGPT and Bard. The results show they can be effective solutions, especially BERTimbau variants, which presented values ≥ 92% in all metrics. F1-scores of 96% and 98% of the BERTimbau-Large and the Bing/GPT-4, respectively, suggest that they are the best models with trade-offs between the precision and recall metrics for both classes.

All these results from the Bing/GPT-4, BERTimbau-Large, and BERTimbau-Base models with values ≥ 90% in all metrics, mainly observing the F1-scores, mean that their precision and recall are balanced with each other and both maintain an excellent level. For the identification of suicidal ideation, the results suggest that these models both correctly detect sentences with suicidal ideation (i.e., individuals at risk) and have a lower possibility of mistakenly classifying a sentence with suicidal ideation as without it. For practical purposes, intelligent systems based on one of these models can be very efficient in identifying at-risk individuals based on what they write.

When compared to other BERT-based models, BERTimbau-Large better distinguished between classes using operating points (OPs) that balance TPs and FPs. Bing/GPT-4 achieved the best AUC among generative LLMs. OPs of the remaining generative LLMs indicated generally lower balance between TPs and FPs.

### Implications

People with suicidal behavior might often use social networks to post texts that contain suicidal ideation traits ^45^^,^^46^. Young people are likely to report suicidal thoughts and suicidal risk factors in digital media, such as blog posts, tweets, instant messages, text messages, and e-mails ^47^. Moreover, some studies show an association between suicidal thoughts expressed online and suicidal behavior and, hence, online logs may be used to identify young people at suicidal risk ^48^^,^^49^. Thus, identifying suicidal ideation in electronic texts using AI technologies represents a promising way to capture manifestations of suicide risk ^50^. This approach can facilitate early detection of suicide risk, thereby empowering mental health professionals to implement just-in-time adaptive interventions ^31^, including via mobile apps ^51^.

Compared to BERT-based models, the results of this study create implications regarding the potential use of LLMs in identifying suicidal ideation in Brazilian Portuguese texts, particularly Bing/GPT-4, which performed best. The results indicate that Bing/GPT-4 and BERTimbau models can identify patients with positive manifestations of suicidal ideation with a high probability of the classification being correct. The error rate is small in classifying individuals without suicidal ideation as people with suicidal ideation or vice versa. In other words, the high recall of the models suggests that they have a low error rate in classifying patients at risk of suicide as not at risk, which could lead to the professional not carrying out an intervention.

### Contributions and comparison with prior work

The application of AI models focused on mental health issues is an emerging research area, leveraging structured ^52^ and unstructured ^53^ data. Several studies have used NLP techniques to detect manifestations of mental disorders in different textual data, including social media posts, interviews, and clinical notes ^54^. Studies show a growing interest in applying AI technologies to identify suicidal ideation, as its early identification is essential to prevent patients’ suicidal attempts and behaviors ^28^^,^^55^. Also, studies have explored LLMs as potential tools in healthcare applications ^26^^,^^29^^,^^56^ and conducted performance evaluations on the different available models ^42^. For example, ChatGPT-4 surpassed human professionals in effectively extracting data concerning ultrasound and operative reports for acute appendicitis ^57^. ChatGPT was also proposed as a support tool for radiologists, assisting in differential diagnosis, facilitating decision-making, and streamlining workflow efficiency ^58^. However, to the best of our knowledge, this is the first comparative analysis study on the performance of BERT-based and large language models in identifying suicidal ideation in Brazilian Portuguese texts.

Levkovich & Elyoseph ^27^ evaluated ChatGPT’s capacity to identify suicide risk in contrast to psychological assessments by mental health professionals. ChatGPT-4 achieved better precision in suicidal ideation recognition, and the results indicated that it estimated the probability of suicide attempts similarly to the assessments provided by professionals. According to the authors, ChatGPT-4 shows the potential to minimize the actual level of suicide risk when applied to support patients and mental health professionals’ decision-making; however, it still requires new experimental research. In our study, Bing, which is based on GPT-4, performed best. Therefore, our results are similar to those found by Levkovich & Elyoseph ^27^.

Recent studies have investigated the performance of LLMs to detect suicide ideation and risk. Bhaumik et al. ^26^ evaluated the performance of the bi-LSTM, ALBERT, Bio-Clinical BERT, ChatGPT-3.5, and an Ensemble model to detect suicidal ideation from the Reddit dataset that contains 232,000 posts in English marked as suicidal or non-suicidal. The dataset is a collection of posts from “SuicideWatch” and “depression” subreddits (i.e., subcommunities) of the Reddit platform. The authors used 200,000 posts to develop the models, and the remaining posts (32,000 posts) were used for evaluation. Similar to our results found for BERTimbau and Bing, all LLMs performed exceptionally well (> 91% for all metrics). ALBERT performed better than all LLMs, including ChatGPT-3.5 with a zero-shot approach. Therefore, in accordance with our study findings, the BERT-based model was superior to ChatGPT-3.5 in detecting suicidal ideation.

Qi et al. ^59^ evaluated the effectiveness of ChatGPT-3.5 and ChatGPT-4 in identifying suicide risk in Chinese texts from Chinese social media platforms using zero-shot and few-shot prompts. Furthermore, the fine-tuning approach was also evaluated in this study, i.e., submitting additional task-specific prompts that enables users to optimize the performance of ChatGPT-3.5. According to the authors, in the task of identifying suicide risk, no statistically significant differences were found between the different prompt approaches. In the few-shot tests, adding more data did not consistently improve the performance of the generative LLMs. Generally, ChatGPT-4 outperformed ChatGPT-3.5. However, this trend was interrupted when ChatGPT-3.5 underwent fine-tuning, outperforming ChatGPT-4. These results suggest that fine-tuning and task-specific instances can significantly change the performance landscape. Therefore, we found a similarity between the findings obtained by Qi et al. ^59^ and ours, as LLMs show potential for use in supporting professionals. Bing/GPT-4 was quite efficient compared to ChatGPT-3.5.

Unlike the studies above, this work investigated the binary classification of non-clinical Brazilian Portuguese texts based on LLMs. Mental health professionals labeled the dataset used. This study advanced the research initiated by Diniz et al. ^21^ by comparing the performance of different BERT-based and large language models in identifying suicidal ideation from non-clinical texts. Finally, this study adheres to the principles of open science, as it presents a good score in the quality assessment.

### Strengths and limitations

We highlight the rigor of the methodology adopted in this study to minimize the risks of bias and ensure a fair evaluation of the models. The dataset was rigorously labeled by psychologists from different paradigms ^21^. We balanced the training data between the two classes to obtain fine-tuned BERT-based models. We found no issues with missing data or features in the dataset. The test data is equally distributed between classes (50 sentences from each class). The models were not tested with synthetically generated sentences. BERT-based models were pre-trained with Brazilian Portuguese texts. Furthermore, we evaluated the models using metrics that aid us discover whether a model performs worse for one class than another, for example, precision and recall. BERT-based models and Bing/GPT-4 do not present class bias issues because the performance of each metric was similar. For ethical reasons, the dataset does not contain information that could identify the users of X (former Twitter) who produced them ^21^.

This study has limitations. First, we did not evaluate different prompting strategies to analyze whether there was a significant difference in the performance of generative LLMs, i.e., we adopted only the zero-shot prompting. Although some studies report no difference in performance between zero-shot and few-shot methods ^42^^,^^59^, evaluating different prompting strategies could allow for in-depth analysis of generative LLMs. Second, our comparison study of different LLMs was limited to the dataset with non-clinical texts classified using binary labels: positive and negative for suicidal ideation. Therefore, a multiclass classification dataset could have been used, i.e., based on ordinal categorical variables to express, for example, levels of suicidal ideation or risk levels for suicide.

## Conclusion

This study demonstrated that LLMs, particularly Bing/GPT-4 and BERTimbau, show potential clinical applicability for identifying suicidal ideation due to their performance results. Our results suggest that intelligent systems based on Bing/GPT4 or BERTimbau can be very efficient in identifying individuals at risk of suicide based on the texts they produce, which might enable just-in-time interventions by mental health professionals.

More research and computational experiments are needed when using LLMs to support mental health professionals in detecting suicidal ideation in Brazilian Portuguese texts, such as varying prompting strategies and analyzing sentiments/emotions related to mental disorders (e.g., depression, anxiety). LLMs are continually evolving, which is reflected in the change of model names (e.g., Bing/GPT-4 is now Microsoft Copilot and Bard became Gemini). As a consequence, the results presented in this study are not final, and further studies may update them as newer solutions become available. Finally, despite the potential of the models in supporting suicidal ideation detection, this study was not validated in a patient monitoring clinical setting. Caution is needed when using the evaluated models, mainly Bard and ChatGPT-3.5, to support mental health professionals in detecting suicidal ideation in Brazilian Portuguese texts. Therefore, follow-up studies are required for all models investigated in this study before their application in clinical settings.

## Data Availability

The dataset used in this study is available on https://doi.org/10.5281/zenodo.10065214. The code that have been used in this study is available on https://github.com/adonias-caetano/Suicidal-Ideation-BERTvsLLM.git.
