# Supplementary material for: Comparative analysis of BERT-based and generative large language models for detecting suicidal ideation: a performance evaluation study
Source: Cad Saude Publica. 2024 Nov 25;40(10):e00028824. doi: 10.1590/0102-311XEN028824 (PMC11654116; doi:10.1590/0102-311XEN028824)
Supplement: Supplementary Material - Table S1 [file 1678-4464-csp-40-10-EN028824-s.pdf]

SUPPLEMENTARY MATERIAL

Table S1 Comparative Analysis of BERT-Based and Large Language Models for Detecting Suicidal Ideation: A Performance Evaluation Study.

Predictions of suicidal ideation (SI) of 100 sentences in Brazilian Portuguese (PT-BR) of the models evaluated: BERTimbau-Base, BERTimbau-Large, BERT-multilingual, Bing/GPT-4, ChatGPT-3.5 and Bard.

Prediction outputs: 0 (Negative for SI), 1 (Positive for SI), and 2 (unknown responses).

Note: The Bard and Bing/GPT-4 chatbots were the only models that gave unknown answers for some sentences. So, there are two columns for these models. A column where these responses were treated as prediction errors and another column to record the original response (prediction output = 2).

SENTENCES IN PT-BR	Actual label	Predicted label							
		BERTimbau-Base	BERTimbau-Large	BERT-multilingual	ChatGPT-3.5	Bard	Bard_unknown	Bing/GPT-4	Bing/GPT-4_unknown
Aquela vontade de acabar com a minha vida voltou	1	1	1	1	1	1	1	1	1
to triste e com vontade de acabar com a minha vida	1	1	1	1	1	1	1	1	1
Corinthians ta querendo acabar com minha vida mesmo	0	0	0	0	0	0	0	0	0
Alguém poderia por favor me dar um tiro a acabar com a minha vida	1	1	1	1	1	1	1	1	1
TAYLOR TU VAI acabar com a minha vida MULHER	0	0	0	0	0	0	0	0	0
Eu vou assistir o jogo todo puto mais uma vez. Esse velho maldito vai conseguir acabar com a minha vida	0	0	0	0	0	1	2	0	0
Alguem pode me.indicar uma série uma boa q vai acabar com a minha vida social pelas próximas semanas??	0	0	0	0	0	0	0	0	0
tenho 76 slides p estudar obg faculdade por acabar com a minha vida social	0	1	0	1	0	0	0	0	0
eu nao concordei com um tweet da fernanda agora ela desenterrou um hit meu pra acabar com a minha vida acho lindo a amizade pós-moderna	0	0	0	0	0	0	0	0	0
sessão de foto pra acabar com a minha vida esse fudido https://twitter.com/h1ghrarchive/status/1117462044226101253 …	0	0	0	0	0	1	2	0	0

quem te deu permissão pra acabar com a minha vida desse jeito seu fdp te amo demais bixo escroto da minha vida todinha <3<3<3	0	0	0	0	0	1	1	0	0
Se eu decidir acabar com minha vida hoje vai ser ótimo pq realmente eu não aguento mais	1	1	1	1	1	1	1	1	1
Galo vai acabar com minha vida acadêmica	0	0	0	0	0	1	2	0	0
Escola vai acabar com minha vida social é isso	0	0	0	0	0	0	0	0	0
Q lindo tem resumo e report de ing e ainda mais slides de artes tudo pra seg sendo é véspera de prova Esses projetos tão tentando acabar com minha vida juro	0	0	0	0	0	1	1	0	0
Esse meu vizinho quer acabar com a minha vida colocando esses pagodes que dói até a alma.	0	0	0	0	0	0	0	0	0
A madonna quer acabar com a minha vida mesmo pic.twitter.com/S23kg7Ocn5	0	0	0	0	0	1	2	0	0
eu não penso em suicídio há uns dois anos mas isso não significa que eu não pense em morrer. eu nunca faria algo para acabar com minha vida mas eu desejo o fim dela frequentemente. é estranho	1	1	1	1	0	1	1	1	1
a única pessoa que eu podia desabafar e de fato me entendia eu consegui deixar escapar e como me disseram isso foi o gatilho que faltava pra me dar coragem de acabar com a minha vida . mas calma. ninguém tem culpa de nada. ninguém além de mim.	1	1	1	1	1	1	1	1	1
esse professor de comunicação meios e significados quer acabar com minha vida	0	0	0	0	0	0	0	0	0
eu ouço que é coisa da minha idade e que vai passar q eu só tô em uma baita crise existencial. SE FOSSE SÓ ISSO EU NÃO ESTARIA NESSE PONTO DE JÁ TER TENTANDO acabar com a minha vida poxa ninguém consegue se colocar no meu lugar? não é impossível.	1	1	1	0	1	1	1	1	1
jungkook você ama acabar com a minha vida né??	0	0	0	0	0	1	1	0	0
Game of Thrones está voltando para acabar com minha vida	0	0	0	0	0	0	0	0	0
Vontade mesmo de mandar tudo pro ar e até mesmo acabar com minha vida	1	1	1	1	1	1	1	1	1
e eu achando que essas fotos não iriam acabar com a minha vida até after 2 pic.twitter.com/y9koF4PRdn	0	0	0	0	0	1	1	0	0
ela vai voltar pra acabar com a minha vida https://twitter.com/taylorswift13/status/1117091141596385280 …	0	0	1	1	0	1	1	0	0
eu hoje falando que a comunidade lgbt+ entrou num padrão e namora pessoas iguais a si e que é comum o preterimento do	0	0	0	0	0	1	1	0	0

negro e a objetificação da mulher negra e etc com um casal gay branco atrás de mim que podia me acusar de homofobia e acabar com a minha vida									
Aliás por isso que eu não assino. Pra não acabar com a minha vida social	0	0	0	0	0	1	1	0	0
eu mando cada foto pro Romulo e pra Maria que se um dia eles quiserem acabar com a minha vida tá facinho	0	0	0	0	0	1	1	0	0
essa conta ainda vai acabar com a minha vida https://twitter.com/badpostandy/status/1117092258636009472 …	0	0	0	0	0	1	1	0	0
Desgraçada fdp fazendo macumbinhaaaa pra acabar com a minha vida achando que eu não ia saber	0	0	0	0	0	1	1	0	0
Mulheres ruivas vão acabar com minha vida	0	0	0	0	0	1	2	0	0
Essa tequila vai acabar com a minha vida kkkkkk	0	0	0	0	0	1	1	0	0
Hj as 21h47 minha mãe me bate me deixando machucada e me ameaça apenas por eu falar que estou namorando uma garota... Sério eu não tô bem eu acho que vou acabar com a minha vida de vez... então... É isso estou machucada mais a morte vai me fazer esquecer isso	1	0	1	0	1	1	1	1	1
nossa outra igual eu nunca vi na vida ela é puro embalo é disparo é de parar a avenida já é pura adrenalina no meu coração essa sensação mina tu vai acabar com a minha vida	0	0	0	0	0	0	0	0	0
O problema é que meu coração sempre fala de mais sai dizendo cada eu te amo pra quem não devia dessa vez ele escolheu você pra acabar com a minha vida outra vez tô colecionando garrafas vazias	0	0	0	0	0	1	1	1	2
pensando em como vou pegar uma barra de chocolate e comer tudo sem minha mãe perceber e acabar com minha vida antes de eu repor	0	0	0	1	0	1	1	0	0
o Bayern querer contratar o Werner é pra querer acabar com a minha vida de vez.	0	0	0	0	0	1	1	0	0
maravilha meu pai vai tirar meu celular de novo e meu psicológico está 100% abalado se eu não acabar com minha vida hj...	1	0	0	0	1	1	1	1	1
Saber que o louco do Bira pode acabar com minha vida com um simples gesto me preocupa	0	1	0	0	0	0	0	0	0
O MEJ quer acabar com a minha vida	0	0	0	1	0	1	2	0	0
Maldito sertanejo pra acabar com a minha vida	0	0	0	0	0	1	2	0	0
mesmo tendo provavelmente umas 3 provas que vão acabar com a minha vida	0	0	0	0	0	1	1	0	0

Assistindo This Is Us pra acabar com a minha vida de vez	0	0	0	1	0	1	1	0	0
Matheus e Kauan para de acabar com minha vida AGORA	0	1	1	1	0	1	1	0	0
O problema é que meu coração sempre fala demais Sai dizendo cada eu te amo Pra quem não devia Dessa vez ele escolheu você Pra acabar com a minha vida	0	0	0	0	0	1	1	0	0
salário caindo em pleno sabadou e hj tem logo o show do turma do pagode vou acabar com a minha vida mais uma vezzzz	0	0	0	0	0	1	1	0	0
Taylor Swift está voltando para acabar com a minha vida	0	0	0	0	0	0	0	0	0
o objetivo de yuri ontem no rolê foi acabar com a minha vida só isso	0	1	0	0	0	0	0	0	0
Eu preciso acabar com minha vida o mais rápido possível	1	1	1	1	1	0	2	1	1
heloísa vai acabar com a minha vida kkkkkkk prevejo	0	0	0	0	0	1	2	0	0
Dessa vez ele escolheu você pra acabar com a minha vida	0	0	1	1	0	1	1	0	0
TAYLOR SWIFT ESTÁ VOLTANDO PARA acabar com a minha vida ! #TS7	0	0	0	1	0	0	0	0	0
A porra dessa uber Eats quer acabar com minha vida social	0	0	0	0	0	1	2	0	0
esss relatório de estágio vai acabar com a minha vida social eu sinto	0	0	0	0	0	0	0	0	0
Se aquele menino não acabar com minha vida .. Não considero ele fdp	0	0	0	1	0	1	2	0	0
nossa estou cansado de viver	1	1	1	1	0	1	1	1	1
Tô tão cansado dessa vida. Tô tão cansado de não conseguir ser feliz de não ver luz no fim do túnel. Tô realmente bem cansado de viver assim.	1	1	1	1	1	1	1	1	1
oficialmente cansado de viver	1	1	1	1	1	1	1	1	1
Primeiro capítulo no ar! "Ian Thompson está cansado de viver a vida que seus pais escolheram para ele e por isso decide voltar para sua cidade natal Charleston e alugar um apartamento próximo a casa do avô... https://www.facebook.com/nataliadonatto/posts/2199185466842273 …	0	0	0	0	0	0	0	0	0
Tou tão cansado de viver .	1	1	1	1	0	1	1	0	2
#FinalBBB19 cansado de viver EM UM PAÍS QUE SÓ ANUNCIA TRAGÉDIAS LIXO	0	0	0	0	0	1	1	0	0
Na Porta ao Lado volta amanhã para o Wattpad e hoje ganhou capa e sinopse novas! "Ian Thompson está cansado de viver a vida que seus pais escolheram para ele e por isso decide voltar para sua cidade natal...	0	0	0	0	0	1	1	0	0

https://www.facebook.com/nataliadonatto/posts/2198165063610980 …									
cansado de viver chega de ser um tormento na vida das pessoas	1	1	1	1	0	1	1	1	1
Oq vc tem? "Nada é só cansaço" Cansaço psicológico... cansado de viver isso sim.	1	1	1	1	0	1	1	1	1
cansado com dor de cabeça e sem vontade de viver	1	1	1	1	0	1	1	1	1
Sabe o que e estar cansado de viver ?? Pse to nesse nivel ja	1	1	1	1	0	1	1	1	1
Realmente tô cansado de viver	1	1	1	1	0	1	1	1	1
Tem como não acordar mais tô cansado de viver isso todo dia	1	1	1	1	1	1	1	1	1
aí namoral? a existência cansa to cansado de viver	1	1	1	1	1	1	1	1	1
Você está triste e cansado de viver a vida em um carrossel E você não pode encontrar o lutador Mas eu vejo isso em você então nós vamos caminhar com isso E mover montanhas Nós vamos caminhar com isso E mover montanhas @AndraDayMusic	0	0	0	0	0	0	0	0	0
cansado de viver dividido entre política e varejo. as urgências são as mesmas e tudo é pra ontem.	0	0	0	0	0	0	0	0	0
Cada vez mais cansado de viver	1	1	1	1	1	1	1	1	1
eu nunca tinha entendido isso. mas agora eu entendo. eu to cansado de viver .	1	1	1	1	0	1	1	1	1
eu to cansado de viver isso qria q tudo acabasse	1	1	1	1	1	1	1	1	1
To cansado de viver mesmo	1	1	1	1	1	0	2	1	1
aff cansado de viver é sério	1	1	1	1	0	1	1	1	1
sei lá só queria dormir pra sempre	1	1	1	1	0	1	1	1	1
daí você mistura um monte de remédios esperando sei lá dormir pra sempre ??? e acorda já no dia seguinte só com uma dor no estômago absurda ???? acordada triste com dor no estômago mais azarada que eu???	1	1	1	0	1	1	1	1	1
cara só queria dormir pra sempre até td isso passar ainda n superei nada	1	1	1	1	0	1	1	1	1
Eu queria dormir pra sempre deve ser mt bom	1	1	1	1	1	1	1	1	1
quero dormir pra sempre tem como?	1	1	1	1	1	0	2	1	1
só quero dormir pra sempre a	1	1	1	1	1	0	2	1	1
só quero dormir pra sempre	1	1	1	1	1	1	1	1	1
To só pensando: Aquele dia a gente acorda com vontade de dormir pra sempre porque tudo está uma bosta	1	1	1	1	0	1	1	1	1

Isso é vdd queria dormir pra sempre	1	1	1	1	1	1	1	1	1
Odeio a minha vida eu só queria dormir pra sempre e nunca acordar	1	1	1	1	1	1	1	1	1
momento que eu não sei o que to sentindo e to sem propósito nenhumqueria dormir pra sempre	1	1	1	1	1	1	1	1	1
que vontade de dormir pra sempre	1	1	1	1	1	1	1	1	1
queria dormir pra sempre sabe	1	1	1	1	1	1	1	1	1
eu me sinto horrível. Eu prometi pra tanta gente que iria tentar melhorar mas as coisas só pioram e pioram. tudo dentro de mim dói de uma maneira insuportável eu nem quero mais que as pessoas entendam. não quero tentar explicar ou melhorar eu só quero dormir pra sempre .	1	1	1	1	1	1	1	1	1
vontade de dormir pra sempre	1	1	1	1	1	1	1	1	1
Áh mano sei la Só queria dormir pra sempre	1	1	1	1	1	1	1	1	1
Só queria dormir pra sempre ..	1	1	1	1	1	1	1	1	1
sou gorda sofri mt bullying e nunca vou conseguir ter uma vida boa porque a crise de ansiedade e depressão tomaram conta de mim só queria dormir pra sempre	1	1	1	1	0	1	1	1	1
To descrente da minha vida angústia sem motivo vontade de dormir pra sempre	1	1	1	1	0	1	1	1	1
Pq ultimamente puf só queria dormir pra sempre	1	1	1	1	0	1	1	1	1
vontade de dormir pra sempre	1	1	1	1	0	1	1	1	1
to cansada quero dormir pra sempre	1	1	1	1	0	0	2	1	1
Cansada demais queria dormir pra sempre	1	1	1	1	0	0	0	1	1

Table S2 Checklist for assessment of requirements and recommendations for sound medical ML contributions to the existing literature. NA: not applicable; mR: minor revisions needed; MR: major revisions needed. Items in bold indicate priority aspects to be considered. Items denoted with a § symbol are directly inspired by the MINIMAR guideline [49]. The section names for the checklist items are directly inspired by the CRISP-DM framework [50].

Available in https://www.sciencedirect.com/science/article/pii/S1386505621001362.

References:

[49] T. Hernandez-Boussard, S. Bozkurt, J.P. Ioannidis, N.H. Shah. Minimar (minimum information for medical AI reporting): developing reporting standards for artificial intelligence in health care. J. Am. Med. Inform. Assoc., 27 (12) (2020), pp. 2011-2015

[50] R. Wirth, J. Hipp Crisp-dm: towards a standard process model for data mining. Proceedings of the 4th International Conference on the Practical Applications of Knowledge Discovery and Data Mining, vol. 1, Springer-Verlag, London, UK (2000)

**Problem understanding**	
Requirement	Answer
**1. Is the study population described, also in terms of inclusion/exclusion criteria (e.g., patients older than 18 tested for COVID-19; all inpatients hospitalized for 24 or more hours)? §**	NA
**2. Is the study design described? (e.g., retrospective, prospective, crosssectional [51], observational, randomized control trial [52]) §**	Yes
**3. Is the study setting described? (e.g., teaching tertiary hospital; primary care ambulatory, nursing home, medical laboratory, R&D laboratory) §**	NA
**4. Is the source of data described? (e.g., electronic specialty registry; laboratory information system; electronic health record; picture archiving and communication system) §**	Yes
**5. Is the medical task reported? (e.g., diagnostic detection, diagnostic characterization, diagnostic staging, prognosis (on which endpoint), event prediction, risk stratification, anatomical structure segmentation, treatment selection and planning, monitoring) §**	Yes
**6. Is the data collection process described, also in terms of setting-specific data collection strategies (e.g., whether body temperatures are measured only in the morning; whether some blood tests are performed only in light of a specific diagnostic hypothesis)? Any consideration about data quality is appreciated, e.g., in regard to completeness, plausibility, and robustness with respect to upcoding or downcoding practices**	Yes
**Data understanding**	
**7. Are the subject demographics described in terms of**<br>**1. average age (mean or median);**<br>**2. age variability (standard deviation (SD) or inter-quartile range (IQR));**<br>**3. gender breakdown (e.g., 55% female, 44% male, 1% not reported); §**<br>**4. main comorbidities;**<br>**5. ethnic group (e.g., Native American, Asian, South East Asian, African, African American, Hispanic, Native Hawaiian or Other Pacific Islander, European or American White);**<br>**6. socioeconomic status?**	NA
**8. If the task is supervised, is the gold standard described? (e.g., "100 manually annotated clinical notes and pain scores recorded in EHR, Death, re-admission and International Classification of Disease (ICD) codes in discharge letters"). In particular, the authors should describe the process of ground truthing described in terms of:**<br>**1. Number of annotators (raters) producing the labels;**<br>**2. Their profession and expertise (e.g., years from specialization or graduation);**	Yes

**3. Particular instructions given to annotators for quality control (e.g., which data were discarded and why);<br>4. Inter-rater agreement score (e.g., Alpha [53], Kappa [54], Rho [17]);<br>5. Labelling technique (e.g., majority voting, Delphi method [55], consensus iteration).**	
**9. In the case of tabular data, are the features described (also in regard to how they were used in the model in terms of categories or transformation)? This description should be done for all, or, in the case that the features exceed 20, for a significant subset of the most predictive features in the following terms: name, short description, type (nominal, ordinal, continuous), and<br>1. If continuous: unit of measure, range (min, max), mean and standard deviation (or median and IQR). Violin plots of some relevant continuous features are appreciated. If data are hematochemical parameters, also mention the brand and model of the analyzer equipment.<br>2. If nominal, all codes/values and their distribution. Feature transformation (e.g., one-hot encoding) should be reported if applied. Any terminology standard should be explicitly mentioned (e.g., LOINC [56], ICD-11 [57], SNOMED [58]) if applied.**	NA
**Data preparation**	
**10. Is outlier detection and analysis performed and reported? If the answer is yes, the definition of an outlier should be given and the techniques applied to manage outliers should be described (e.g., removal through application of an Isolation Forest model).**	NA
**11. Is missing-value management described? This description should be reported in the following terms:<br>1. The missing rate for each feature should be reported;<br>2. The technique of imputation, if any, should be described, and reasons for its choice should be given. If the missing rate is higher than 10%, a reflection about the impact on the performance of a technique with respect to others would be appreciable [59].**	NA
**12. Is feature pre-processing performed and described? This description should be reported in terms of scaling transformations (e.g., normalization, standardization, log-transformation) or discretization procedures applied to continuous features, and encoding of categorical or ordinal variables (e.g., one-hot encoding, ordinal encoding).**	Yes
**13. Is data imbalance analysis and adjustment performed and reported? The authors should describe any imbalance in the data distribution, both in regard to the target (e.g., only 10% of the patients were affected by a given disease); and in regard to important predictive features (e.g., female patients accounted for less than 10% of the total cases). The authors should also report about any technique (if any) applied to adjust the above mentioned imbalances (e.g., under- or over-sampling, SMOTE).**	Yes
**Modeling**	
**14. Is the model task reported? (e.g., binary classification, multi-class classification, multi-label classification, ordinal regression, continuous regression, clustering, dimensionality reduction, segmentation) §**	Yes
**15. Is the model output specified? (e.g., disease positivity probability score, probability of infection within 5 days, postoperative 3-month pain scores) §**	Yes
**16. Is the model architecture or type described? (e.g., SVM, Random Forest, Boosting, Logistic Regression, Nearest Neighbors, Convolutional Neural Network)**	Yes
**Validation**	
**17. Is the data splitting [60] described (e.g., no data splitting;, k-fold cross-validation (CV); nested k-fold CV; repeated CV; bootstrap validation; leave-one-out CV; 80%/10%10% train/validation/test)? In the case of data splitting, the authors must explicitly state that splitting was performed before any pre-processing steps (e.g., normalization, standardization, missing value imputation, feature selection) or model construction steps (training, hyper-parameter optimization), so to avoid data leakage [61] and overfitting**	Yes
**18. Is the model training and selection described? In particular, the training procedure, hyper-parameter optimization or model selection should be described in terms of<br>1. Range of hyper-parameters [62];<br>2. Method used to select the best hyper-parameter configuration (e.g., Hyper-parameter selection was performed through nested k-fold CV based grid search);<br>3. Full specification of the hyper-parameters used to generate results [62];<br>4. Procedure (if any) to limit over-fitting, in particular as related to the sample size [25].**	Yes
**19. (classification models) Is the model calibration described? If the answer is yes, the Brier score should be reported, and a calibration plot should be presented [63]**	No
**20. Is the internal/internal-external model validation procedure described [60], [64] (e.g., internal 10-fold CV, time-based cross-validation)? The authors should explicitly specify that the sets have been splitted before normalization, standardization and imputation, to avoid data leakage [61] (also refer to item 17 of this guideline). If possible, the authors should also comment on the adequacy of the available sample size for model training and validation [65], [25]. Moreover, the authors should try to choose the test set so that it is the most diverse with respect to the remainder of the sample [66] (w.r.t. some multivariate similarity function) and how this choice relates to conservative (and lower-bound) estimates of the model's accuracy (and performance)**	Yes
**21. Has the model been externally validated [67]? If the answer is yes, the characteristics of the external validation set(s) should be described. For instance, the authors could comment about the**	No

heterogeneity of the data with respect to the training set (e.g., degree of correspondence Ψ [66], Data Representativeness Criterion [68]) and the cardinality of the external sample [69]. If the performance on external datasets is found to be comparable with (or better than) that on training and internal datasets, the authors should provide some explanatory conjectures for why this happened (e.g., high heterogeneity of the training set, high homogeneity of the external dataset)	
22. Are the main error-based metrics used?<br>1. a. Classification performance should be reported in terms of: Accuracy, Balanced accuracy, Specificity, Sensitivity (recall), Area Under the Curve (if the positive condition is extremely rare – as in case of stroke events – authors could consider the "Area under the Precision-Recall Curve" [70]). Optionally also in terms of: positive and negative predictive value, F1 score, Matthew coefficient [71], F score of sensitivity and specificity, the full confusion matrix, Hamming Loss (for multi-label classification), Jaccard Index (for multi-label classification).<br>2. Regression performance should be reported in terms of: R2; Mean Absolute Error (MAE); Root Mean Square Error (RMSE); Mean Absolute Percentage Error (MAPE) or the Ratio between MAE (or RMSE) and SD (of the target);<br>3. Clustering performance should be reported in terms of: External validation metrics (e.g., mutual information, purity, Rand index), when ground truth labels are available, and Internal validation metrics (e.g., Davies-Bouldin index, Silhouette index, Homogeneity). The reported results of internal validation metrics should be discussed [72]<br>4. Image segmentation performance, depending on the specific task, should be reported in terms of metrics like [73]: accuracy-based metrics (e.g., Pixel accuracy, Jaccard Index, Dice Coefficient), distance-based metrics (e.g., mean absolute, or maximum difference), or area-based metrics (e.g., true positive fraction, true negative fraction, false positive fraction, false negative fraction).<br>5. Reinforcement learning performance, depending on the specific task, should be reported in terms of metrics like [74]: Fixed-Policy Regret, Dispersion across Time, Dispersion across Runs, Risk across Time, Risk across Runs, Dispersion across Fixed-Policy Rollouts, Risk across Fixed-Policy Rollouts.<br><br>The above estimates should be expressed, whenever possible, with their 95% (or 90%) confidence intervals (CI), or with other indicators of variability [36], with respect to the evaluation metrics reported. In this case, the authors should report which methods were applied for the computation of the confidence intervals (e.g., whether k-fold CV or bootstrap was applied, normal approximation). When comparing multiple models, the authors should discuss the statistical significance of the observed differences [75] (e.g., through CI comparisons, or hypothesis testing). When comparing multiple regression models, a Taylor diagram [76] could be reported and discussed.	Yes
23. Are some relevant errors described? The authors should describe the characteristic of some noteworthy classification errors or cases for which the regression prediction was much higher (>2×) than the MAE. If these cases represent statistical outliers for some covariates, the authors should comment on that. To detect relevant cases, the authors could focus on those cases on which the inter-rater agreement (either re ground truth or by comparing human vs. model's performance) is lowest.	NA
**Deployment**	
24. Is the target user indicated? (e.g., clinician, radiologist, hospital management team, insurance company, patients) §	Yes
25. (classification models) Is the utility of the model discussed? The authors should report the performance of a baseline model (e.g., logistic regression, Naive Bayes). Additionally, the authors could report the Net Benefit [77] or similar metrics and present utility curves [78]. In particular, the authors are encouraged to discuss the selection of appropriate risk thresholds [79]; the relative value of benefits (true positives/negatives) and harms (false positives/negatives); and the clinical utility of the proposed models [25].	Yes
26. Is information regarding model interpretability and explainability available [80] (e.g., feature importance, interpretable surrogate models, information about the model parameters)? Claims of "high" or "adequate" model interpretability (e.g., by means of visual aids like decision trees, Variable Importance Plots or Shapley Additive Exlanations Plots (SHAP) [81]) or model causability [82] should always be supported by some user study, even qualitative or questionnaire-based [83]. In the case surrogate models were applied, the authors should report about their fidelity [84], [85]	No
27. Is there any discussion regarding model fairness, ethical concerns or risks of bias [25], [86] (for a list of clinically relevant biases, refer to [87])? If possible, the authors should report the model performance stratified for particularly relevant population strata [88] (e.g., model performance on male vs. female subjects, or on minority groups)	Yes
28. Is any point made about the environmental sustainability of the model, or about the carbon footprint [89], of either the training phase or inference phase (use) of the model? If the answer is yes, then such a footprint should be expressed in terms of carbon dioxide equivalent (CO2eq) and details about the estimation method should be given. Any efforts to this end will be appreciated, including those based on tools available onlinea, as well as any attempts to popularise this concept, e.g., through equivalences with the consumption of everyday devices such as smartphones or kilometres travelled by a fossil-fuelled carb	NA
29. Is code and data shared with the community [62], [90]? § If not, are reasons given? If code and data are shared, institutional repositories such as Zenodo should be preferred to private-owned repositories (arxiv, GitHub). If code is shared, specification of dependencies should be reported and a clear distinction between training code and evaluation code should be made. The authors should also state whether the developed system, either as a sand-box or as fully-operating system, has been made freely accessible on the Web.	Yes
30. Is the system already adopted in daily practice? If the answer is yes, the authors should report on where (setting name) and since when. Moreover, appreciated additions would regard: the description on the digitized workflow integrating the system; any comment about the level of use [25]; a qualitative assessment of the level of efficacy of the system's contribution to the clinical process (e.g., [91], [92]); any comment about the technical and staff training effort actually required [25]. If the answer is no, the authors should be explicit in regard to the point in the clinical workflow where the ML model should be applied, possibly using standard notation (e.g., BPMN). Moreover, the authors should also propose an assessment of the technology readiness of the described system, with explicit reference to the Technology Readiness Level frameworkc or to any adaptation of this framework to the AI/ML domain [93]. In either above cases (yes/no), the authors should report about the procedures	NA

**(if any) for performance monitoring, model maintenance and updating [94].**	
